# Newly Developed Correlations to Predict the Rheological Parameters of High-Bentonite Drilling Fluid Using Neural Networks

**DOI:** 10.3390/s20102787

**Published:** 2020-05-14

**Authors:** Ahmed Gowida, Salaheldin Elkatatny, Khaled Abdelgawad, Rahul Gajbhiye

**Affiliations:** Petroleum Department, College of Petroleum Engineering & Geosciences, King Fahd University of Petroleum & Minerals, Dhahran 31261, Saudi Arabia; g201708730@kfupm.edu.sa (A.G.); abouzidan@kfupm.edu.sa (K.A.); rahulg@kfupm.edu.sa (R.G.)

**Keywords:** rheological properties, high-bentonite mud, artificial neural network, mud weight, marsh funnel

## Abstract

High-bentonite mud (HBM) is a water-based drilling fluid characterized by its remarkable improvement in cutting removal and hole cleaning efficiency. Periodic monitoring of the rheological properties of HBM is mandatory for optimizing the drilling operation. The objective of this study is to develop new sets of correlations using artificial neural network (ANN) to predict the rheological parameters of HBM while drilling using the frequent measurements, every 15 to 20 min, of mud density (MD) and Marsh funnel viscosity (FV). The ANN models were developed using 200 field data points. The dataset was divided into 70:30 ratios for training and testing the ANN models respectively. The optimized ANN models showed a significant match between the predicted and the measured rheological properties with a high correlation coefficient (R) higher than 0.90 and a maximum average absolute percentage error (AAPE) of 6%. New empirical correlations were extracted from the ANN models to estimate plastic viscosity (PV), yield point (Y_P_), and apparent viscosity (AV) directly without running the models for easier and practical application. The results obtained from AV empirical correlation outperformed the previously published correlations in terms of R and AAPE.

## 1. Introduction

Drilling Fluids play a pivotal role during the drilling operation [1]. There are three main categories of the drilling fluid, namely water-based mud, oil-based mud, and synthetic-based mud, used to enhance the drilling operation performance under downhole conditions of pressure and temperature [2]. The main function of a drilling fluid is to clean the wellbore by lifting the drilled cuttings from the bottom of the hole up to the surface; then the cuttings are treated by the solid control equipment before being pumped again into the well [3]. Special viscous mud (known as spud mud) is commonly used while drilling surface sections to help remove large cuttings out of the drilled hole [4]. Moreover, it enhances wellbore stability by forming an impermeable filter cake and minimize fluid loss by stopping mud filtration [5].

### 1.1. High-Bentonite Mud (HBM)

HBM is a certain type of spud mud, which contains high-bentonite concentration. Generally, bentonite is used in drilling fluids to increase its viscosity and provide more colloidal solids, which form an impermeable filter cake and reduces [6]. Increasing the plastic viscosity of drilling fluid leads to a remarkable improvement in cutting removal and hole cleaning efficiency; however, it should be optimized to avoid many drilling problems, such as pipe sticking [7]. HBM mainly comprises of a large amount of pre-hydrated bentonite, ranging from 40 to 50 sacks (2000 Ib-sack) per drill-water barrel to avoid severe flocculation, in addition to acting as a viscosifier and fluid loss control [8]. It also contains enviro-thin as a dispersant to disperse a large amount of bentonite within the fluid and reduce the tendency of mud to coagulate into a mass of particles [9]. Besides, it contains caustic soda NaOH for pH control and a fluid loss additive like starch [10]. The additives used to formulate HBM and their uses are summarized in Table 1. 

### 1.2. Drilling Fluid Rheology

Rheology of drilling fluid is the controlling factor for enhancing the hole cleaning efficiency and optimizing the drilling performance [11,12]. These rheological properties include plastic viscosity (PV), yield point (Y_P_), and apparent viscosity (AV) for evaluating the mud performance during drilling operation [13]. 

Plastic viscosity (PV) indicates the amount of solids existing in the drilling fluid [14]. Uncontrolled increase of the mud solid content may lead to many critical problems while drilling like pipe sticking and reducing the rate of penetration [15]. Yield point is another rheological parameter measuring the attractive forces among colloidal particles within the drilling fluid [14]. Optimizing Y_P_ significantly affects hole-cleaning efficiency [15].

Mud rheological properties are experimentally estimated using conventional rheometer and mud balance. The rheometer can be simply described as a coaxial cylindrical rotational viscometer. During the measurement of the rheological properties of the drilling fluid using rheometer, the drilling fluid is contained in the annular space or the shear gap between the cylinders. Then the viscosity is determined based on the measurements of applied shear rate and the corresponding shear stress at different rotation speeds. More details on the rheometer design and the measuring technique are described in [16,17]. Common field practice comprises only measuring mud density by mud balance and mud viscosity by Marsh funnel periodically every fifteen minutes to monitor any changes in the rheology of the drilling fluid. A complete mud test (including all mud rheological properties) is performed twice a day since it consumes considerable time. 

In 1960, Marsh funnel viscosity (FV) was introduced to indicate the changes in the rheology of the drilling fluid and measured by the Marsh funnel device. This tool is effectively practical because it takes a short operating time and can be utilized to frequently measure FV [18]. 

Some empirical models have been developed to determine rheological parameters of the drilling fluid using Marsh funnels. Some of the proposed models monitor the change in the mud height in Marsh funnel with time and correlate it with the fluid rheological properties such as PV, Y_P_, and AV [19,20,21,22]. The shear rate and the shear stress are estimated on the sides of the Marsh funnel using the volume of the mud coming out at different points. The measured shear rate and shear stress are then correlated to the rheological parameters. The Marsh funnel was used to investigate several water-based drilling fluids and it was proved that both PV and AV can be estimated using consistency plots [23]. Results from these models were extremely different from the measurements of the Marsh funnel and conventional rheometer. Other trials were conducted for accuracy improve by using polynomial functions of a high order to model the fluid flow volume through the marsh funnel instead of the simple equations used before [24,25]. These trials accurately simulated the change in fluid height within the Marsh funnel with time and achieved better estimations of the rheological parameters compared to those obtained from the standard rheometer.

The main objective of this study is to identify the rheological flow model of HBM experimentally and develop new sets of correlations using artificial neural networks to estimate the rheological parameters of HBM from available.

### 1.3. Predicting the Rheological Properties of Drilling Fluid While Drilling 

Periodic monitoring of the parameters controlling the drilling operation is crucial for improving the drilling performance and avoiding any drilling problems. Therefore, hole cleaning and bit hydraulics should be optimized [26]. Optimization of drilling hydraulics accounts for the pressure losses, which depends mainly on the rheological properties of the drilling fluid used. Pressure losses through the annulus can be determined once the parameters of the Bingham plastic model (Y_P_ and PV) are known using Equation (1), assuming laminar flow [27]. Moreover, equivalent circulating density (ECD) can be estimated using Equation (2), which represents the apparent mud weight in dynamic conditions. ECD accounts for many drilling problems like loss of circulation and well control incidents.

(1) $$\Delta P=\left[\frac{\mathrm{PV}\times v}{1000{\left(d_{2}-d_{1}\right)}^{2}}+\frac{\mathrm{YP}}{200\left(d_{2}-d_{1}\right)}\right]L$$

(2) $$\mathrm{ECD}=\mathrm{MD}+\frac{\Delta P}{0.052\times h}$$

where ΔP is the pressure losses through the annulus (psi), PV is the plastic viscosity of the drilling fluid (cP), v is the average velocity (ft/s), Y_P_ is the yield point (lb/100ft^2^), $d_{1}$ is hole diameter (in), $d_{2}$ is the drill pipe outer diameter (in), L is the annulus length (ft), ECD is the equivalent circulation density (lb/ft^3^), MD is the mud density (lb/ft^3^), and h is true vertical depth (ft).

Furthermore, surge and swab pressures can be estimated using Equation (1); after replacing the value of the average velocity (v) in Equation (1) with the effective velocity ($v_{e}$), which can be calculated using Equation (3) [28].

(3) $$v_{e}=v_{m}-kv_{p}$$

where $v_{m}$ is the mud velocity (ft/s), $v_{p}$ is the pipe velocity (ft/s), k is the clinging constant.

## 2. Methodology

### 2.1. Experimental Work 

The rheological models are critical for simulating the characteristics of drilling mud under dynamic conditions to determine key parameters such as equivalent circulating density, pressure drop, hole cleaning efficiency. All of these parameters are required to design and evaluate the hydraulics and assess the functionality of the mud system [29]. There are three well-known mathematical models used to describe the mud rheology; Power Law model, Bingham Plastic model, and Hershel Buckley Model [30,31]. Each model has specific parameters to describe the drilling fluid performance such as shear stress, shear rate, flow behavior index, and consistency coefficient. To study the rheological behavior of HBM and identify the most appropriate rheological model that follows, mud samples were prepared based on the formulation listed in Table 2.

**Figure 1:**
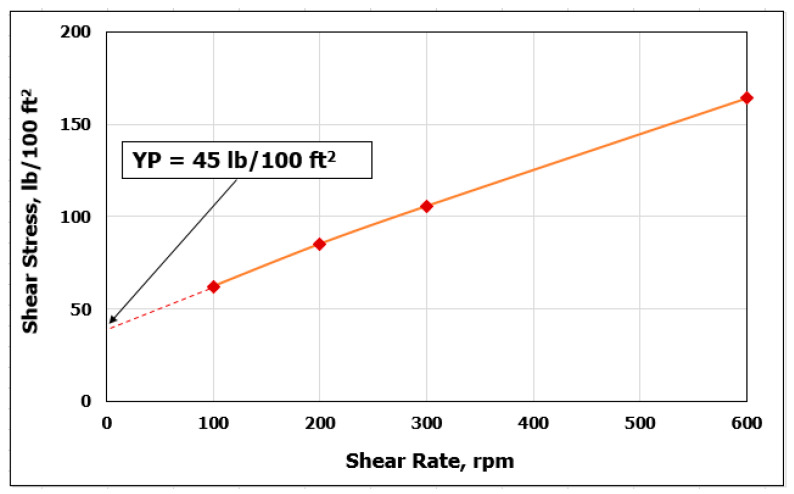
Shear rate vs. shear stress for HBM at 120 °F and atmospheric pressure.

The standard Rheometer was operated at different shear rates and the corresponding shear stress was recorded at 120 °F and atmospheric pressure. The results presented in Figure 1 shows the relation between the shear rate and shear stress for HBM. HBM was believed to follow Bingham plastic behavior due to the linear relationship found between shear stress and shear rate [32]. Mud exhibiting Bingham plastic behavior need shear stress that is higher than a critical value, called the yield point (Y_P_), to start flowing. Once the yield point is reached, changes in shear stress and shear rate are directly proportional. The slope of the curve gives the plastic viscosity (PV) [33]. Based on this result, the behavior of HBM can be described by the two-parameter in the Bingham plastic model, PV and Y_P_. Bingham plastic model does not accurately predict fluid flow behavior at low shear rates’ therefore, only high shear rates are considered in Figure 1. However, it is useful for continuous monitoring and controlling of the drilling fluids’ performance [34].

### 2.2. Implementation of Artificial Neural Network (ANN) to Predict HBM Rheology

ANN is an artificial intelligence (AI) powerful tool that can imitate different complex problems which cannot be treated using conventional regression techniques. Without defining the physics behind the studied phenomenon, ANN can analyze its characteristics [35]. ANN processes the data through a network that mimics biological neural systems [36]. Artificial neurons are the elementary units in ANN. An ANN model consists of three fundamental layers: input layer, hidden layers, and an output layer. These layers are connected and processed with special training algorithm and transfer functions to represent the nature of the problem [35]. The neurons existing in each layer are linked by weighted connections called weights and bias [37]. The output layer is commonly assigned to an activation function of ‘‘pure linear” while there are many available options for the transfer functions assigned to hidden layers such as log-sigmoidal and tan-sigmoidal types [38]. Recently, AI has been widely used in the area of drilling fluid [39]. Some of these applications are drilling optimization [40], optimizing drilling hydraulics [41], and prediction of rheological properties of invert emulsion mud, KCl water-based mud, CaCl_2_ drilling fluid, NaCl water-based drill-in fluid rheological properties [42,43,44,45]. Additionally, new systems were developed using the integration between sensitive sensors measurements and AI application to estimate rheological parameters of non-Newtonian fluids [46]. Furthermore, an automated Marsh funnel was developed using data-driven sensors to allow real-time measurement of FV [47]. Therefore, integration between the developed models and such automated funnel would lead to a complete real-time monitoring system for estimating the rheological properties of the drilling fluid while drilling. 

#### 2.2.1. Data Description

Field measurements (200 data points) include MD, FV, Y_p_, PV, and AV for HBM were collected from field measurements that follow the recommended practice for field testing drilling fluids by API RP 13B-1 [48]. The HBM data represented HBM prepared using the same formulation and mixed with the same service company. During field measurements, the rheometer was used to measure the shear stresses at shear rates of 300 and 600 donated by R600 and R300, respectively. These readings were used to estimate Yp, PV, and AV using Equations (4)–(6), respectively [49,50]. Mud density was measured using a mud balance device. MD and FV measurements were conducted at 80 °F, which is the average surface temperature of the region in which the field under study exists. Therefore, it is recommended to use the developed model for fields within the same surface temperature.

(4) $$\mathrm{PV}=R_{600}-R_{300}$$

(5) $$Y_{P}=\mathrm{PV}-R_{300}$$

(6) $$\mathrm{AV}=\frac{R_{600}}{2}$$

**Figure 2:**
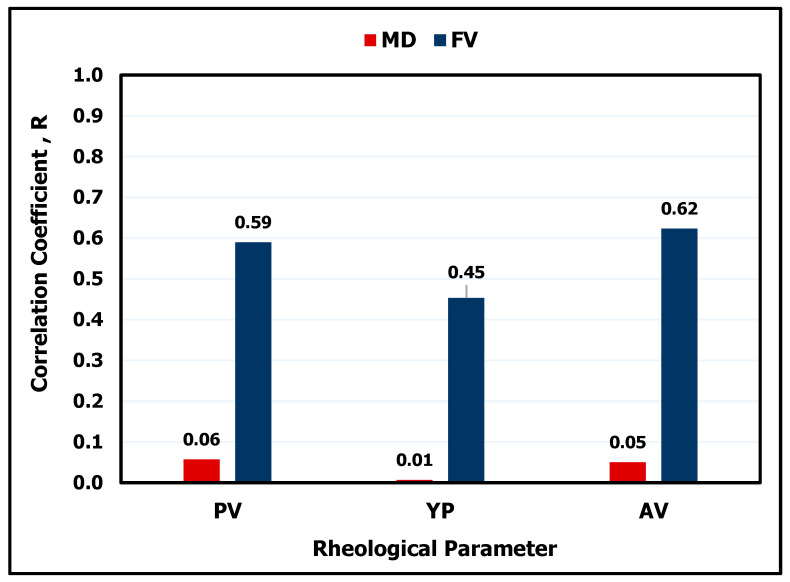
Correlation coefficient (R) between the inputs: mud density (MD) and Marsh funnel viscosity (FV) and the outputs: yield point (Y_P_), plastic viscosity (PV) and apparent viscosity (AV).

The MD ranges from 64 to 73 lb/ft^3^, FV ranges from 45 to 150 s/quart, PV ranges from 11 to 56 cP, YP ranges from 20 to 46 lb/100 ft^2^, and AV ranges from 23 to 79 cP. MD has a low correlation coefficient (R) with $Y_{p}$, PV, and AV, 0.06 at maximum as shown in Figure 2. On the other hand, FV has a correlation coefficient (R) of 0.45, 0.59, and 0.62 with Y_P_, PV, and AV respectively. This higher R-value between the rheological properties with FV compared to MD can be explained as HBM is characterized by its high content of bentonite, which mainly affects the mud viscosity, not the mud weight. Table 3 lists different statistical parameters for the HBM rheological data used in building the ANN models, while Table 4 lists a sample of the obtained data used for training the networks. 

#### 2.2.2. Quality Check and Data Filtration

The higher the quality of the training data is, the better the accuracy of AI models [51]. Thus, the obtained dataset quality was checked using both statistical and technical tools. Unrealistic values like negative and zero values were removed. Then outlier values which show significant deviation from the normal trend of the data were eliminated using the box and whisker plot method [52]. This method comprises two limits (top and bottom) called whiskers representing the upper and lower limits of the data [53]. Values exceeding these two whiskers are considered outliers thus would be removed. These whiskers can be determined using some statistical parameters such as the minimum, maximum, mean, and median parameters (listed in Table 3). According to the reference ranges of the rheological properties of the HBM formulation listed in Table 5, it is clear that the collected data for building the model covers a wide range of these properties, which gives a promising indication on the distribution and the quality of the obtained models.

#### 2.2.3. Model Development 

The collected data were used for building the proposed ANN models. For optimizing the developed models, several scenarios were tested including varying ANN parameters. This was achieved using a specially designed MATLAB code to test all the possible combinations between these parameters. For each scenario (parameters’ combination), the accuracy of the results was evaluated based on the calculated average absolute percentage error (AAPE), in addition to the correlation coefficient (R), to determine how close the predicted values were to the actual values. The varying ANN parameters and their tested ranges were as follows:

– Number of hidden layers (ranges from one to four layers)
– Number of neurons in each layer (range 5: 30 neurons for each layer)
– Transfer functions (tansig, logsig, elliotsig, radbas, satlin, purelin, tribas, hardlim)
– Training algorithm (trainlm, trainrp, traingd, traingda, trainbr, trainc)
– Learning rate (ranges from 0.01 to 0.9)

**Figure 3:**
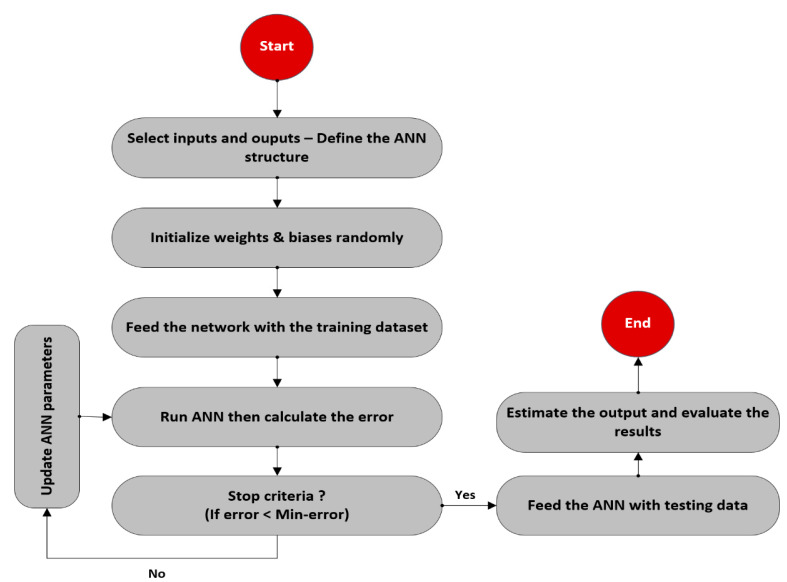
Flowchart describing the workflow of developing the ANN models.

**Figure 4:**
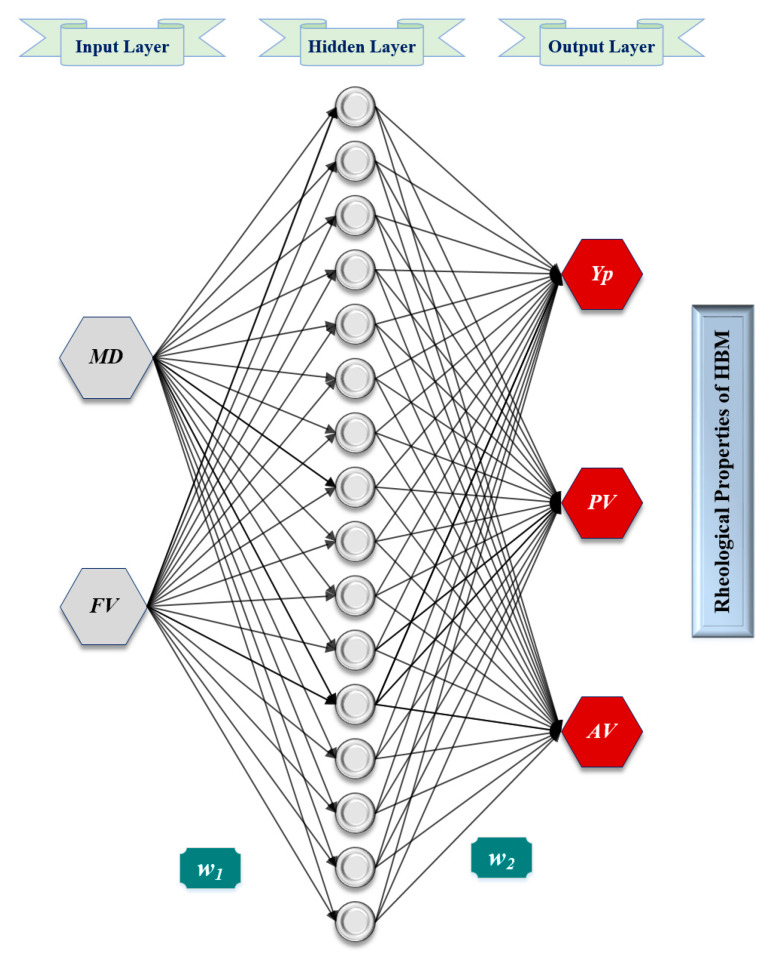
Typical schematic structure for the developed ANN models for predicting the rheological properties of HBM.

Thereafter, the tested parameters, which resulted in the most accurate results indicated by the lowest AAPE and highest correlation coefficient between the predicted and actual values, were selected. The optimization process followed is schematically described in the flowchart shown in Figure 3. The optimized parameters were found to be a single hidden layer with 20 neurons, Levenberg-Marquardt backpropagation (trainlm) training algorithm with a learning rate of 0.12, a tan-sigmoidal transfer function between the input and hidden layers in addition to a pure-linear transfer function between the hidden and output layers. Figure 4 shows the schematic structure of the developed ANN models. 

## 3. Results and Discussion

### 3.1. Yield Point Prediction

**Figure 5:**
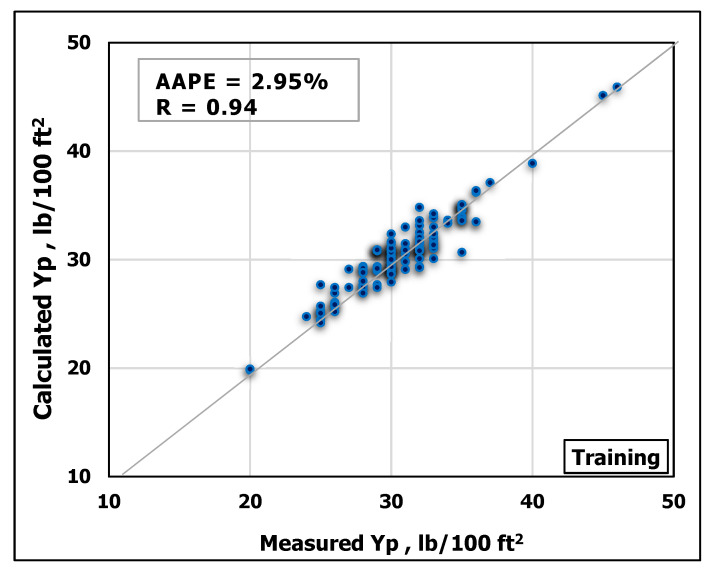
Measured Y_P_ vs. predicted Y_P_ from the ANN model for the training process.

**Figure 6:**
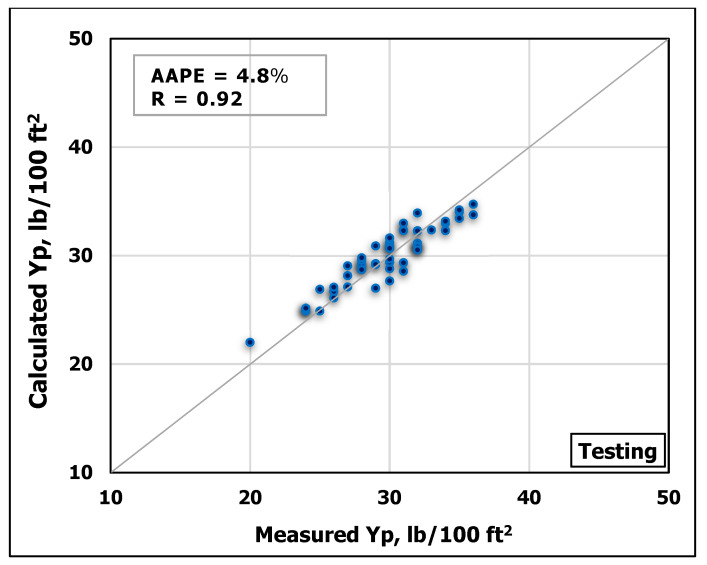
Measured Y_P_ vs. predicted Y_P_ from the ANN model for the testing process.

ANN model was developed using MD and FV as inputs to predict Y_P_. The obtained data were randomly divided using MATLAB program into ratios of 70 percent for training and 30 percent for testing. Figure 5 and Figure 6 show a good match between the predicted Y_P_ values and the actual ones. The high accuracy of the developed model can be inferred from the high R-value of 0.94 for the training process and 0.92 for the testing process in addition to the low AAPE of 2.95% and 4.8% for the training and testing respectively. 

Thereafter, a new correlation was developed using the ANN model to predict Y_P_ based on MD and FV. The developed correlation can be used as follows. First, the inputs should be normalized as described in Appendix A. Then, the normalized value of the output ($Y_{p_{n}}$) is calculated using Equation (7) with its optimized coefficients listed in Table A1 (Appendix B).

(7) $${\mathrm{Yp}}_{n}=\left[\sum_{i=1}^{N}w_{2_{i}}\left(\frac{2}{1+\exp\left(-2\left({\mathrm{MD}}_{n}\times w_{1_{i,1}}+{\mathrm{FV}}_{n}\times w_{1_{i,2}}+b_{1,i}\right)\right)}-1\right)\right]+b_{2}$$

where; (i) is the index of each neuron in the hidden layer, (N) is the optimized number of neurons in the hidden layer, (w_1_) is the weight vector linking the input and the hidden layer, (w_2_) is the weight vector linking the hidden and output layer, (b_1_) is the biases vector for the input layer, (b_2_) is the biases vector for the output layer. For example, $w_{1_{i,1}}$ represents the weight [associated with the neuron of index (i) in the first layer], which would be multiplied by the normalized value of the first input (MW_n_) and similarly $w_{1_{i,2}}$ represents the weight [associated with neuron of index (i) in the first layer], which would be multiplied by the normalized value of the second input (FV_n_). The required Y_P_ value can then be obtained by denormalizing $Y_{p_{n}}$ using Equation (8).

(8) $$Y_{P}=13\left(Y_{P_{n}}+1\right)+20$$

### 3.2. Plastic Viscosity Prediction

**Figure 7:**
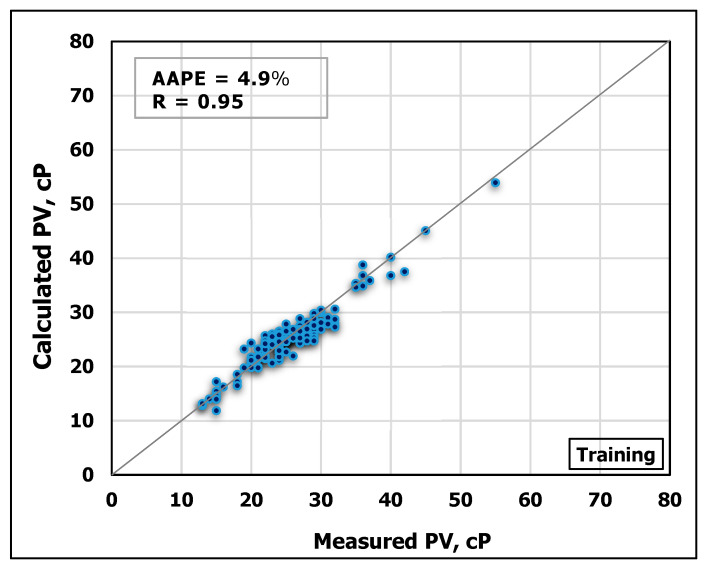
Measured PV vs. predicted PV from the ANN model for the training process.

**Figure 8:**
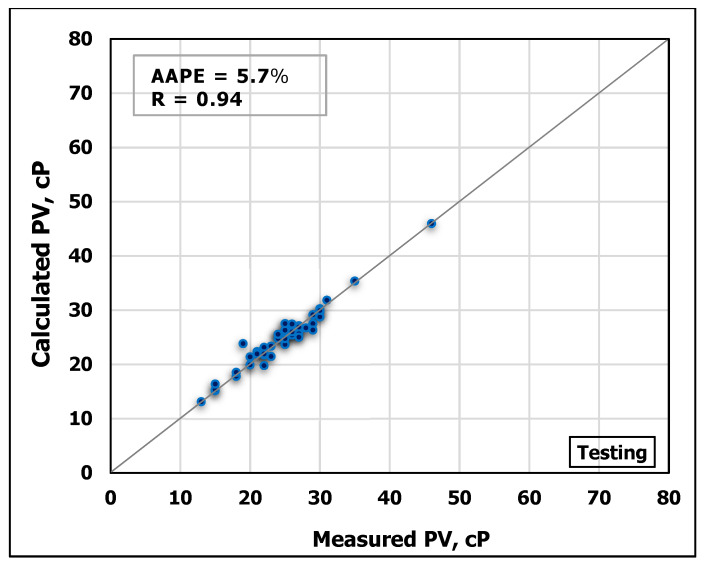
Measured PV vs. predicted PV from the ANN model for the testing process.

Similarly, MD and FV were used to predict PV using ANN. The model was trained using 70% of the obtained data while 30% of the data for testing the model performance. Figure 7 and Figure 8 show cross-plots indicating the high match between the measured and the predicted 

PV values from the developed ANN model. The high accuracy of the developed model can be pointed out from the high R-value of 0.95 for training and 0.94 for testing in addition to low AAPE of 4.9% and 5.7% for training and testing, respectively.

Afterwards, an empirical equation was obtained from the developed ANN model to calculate PV from MD and FV. The normalized PV_n_ was first calculated using Equation (9) with the optimized weights and biases listed in Table A2 (Appendix B). MD_n_ and FV_n_ are the normalized input parameters following the procedures described in Appendix A.

(9) $${\mathrm{PV}}_{n}=\left[\sum_{i=1}^{N}w_{2_{i}}\left(\frac{2}{1+\exp\left(-2\left({\mathrm{MD}}_{n}\times w_{1_{i,1}}+{\mathrm{FV}}_{n}\times w_{1_{i,2}}+b_{1,i}\right)\right)}-1\right)\right]+b_{2}$$

The denormalized value for the output (PV) was finally calculated from the normalized value (PV_n_) using Equation (10).

(10) $$\mathrm{PV}=22.5\left({\mathrm{PV}}_{n}+1\right)+11$$

### 3.3. Apparent Viscosity Prediction

**Figure 9:**
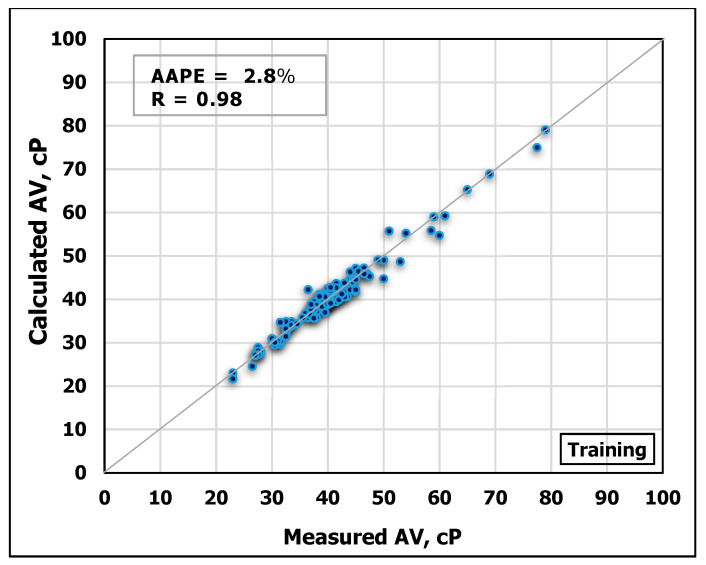
Measured AV vs. Predicted AV from the ANN model for the training process.

**Figure 10:**
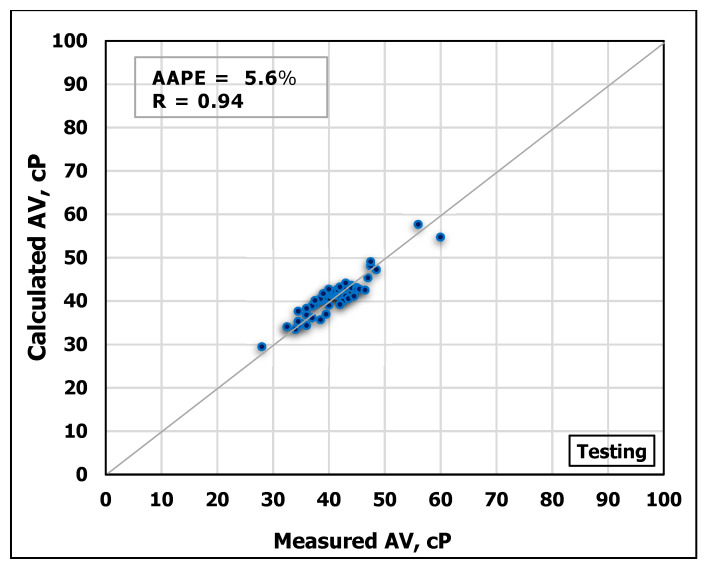
Measured AV vs. Predicted AV from the ANN model for the testing process.

Another ANN model was developed to estimate AV based on MD and FV. The obtained data are partitioned into 70/30 ratios for training and testing the model, respectively. Figure 9 and Figure 10 show the high agreement between the measured and the predicted AV values from the developed ANN model as shown in the cross-plots. The high accuracy of the developed model can be confirmed from the high R-value of 0.98 for training and 0.92 for testing, in addition to the low AAPE of 2.8% and 5.6% for training and testing processes, respectively.

Following that, the empirical correlation was extracted from the developed model to calculate AV directly from MD and FV without the need to run the model. The normalized value AV_n_ would first be calculated using Equation (11), the needed weights and biases for this equation are listed in Table A3 (Appendix B).

(11) $${\mathrm{AV}}_{n}=\left[\sum_{i=1}^{N}w_{2_{i}}\left(\frac{2}{1+\exp\left(-2\left({\mathrm{MD}}_{n}\times w_{1_{i,1}}+{\mathrm{FV}}_{n}\times w_{1_{i,2}}+b_{1,i}\right)\right)}-1\right)\right]+b_{2}$$

Finally, AV can be estimated using Equation (12).

(12) $$\mathrm{AV}=28\left({\mathrm{AV}}_{n}+1\right)+23$$

### 3.4. Apparent Viscosity Model Validation

Based on the literature, two general models were developed to evaluate the mud rheology using mud weight and Marsh funnel viscosity. One was introduced by Pitt [54] to predict the apparent viscosity from mud density and Marsh funnel measurements, as stated in Equation (13). Later, the previous model was modified by Almahdawi et al. [55] as shown in Equation (14), yielding more accurate results compared to Equation (13).

(13) AV=D(T−25)

(14) AV=D(T−28)

where AV is the apparent viscosity of the drilling fluid (cP), and the D was the density of mud (g/cm^3^), and T is the Marsh funnel viscosity (s).

**Figure 11:**
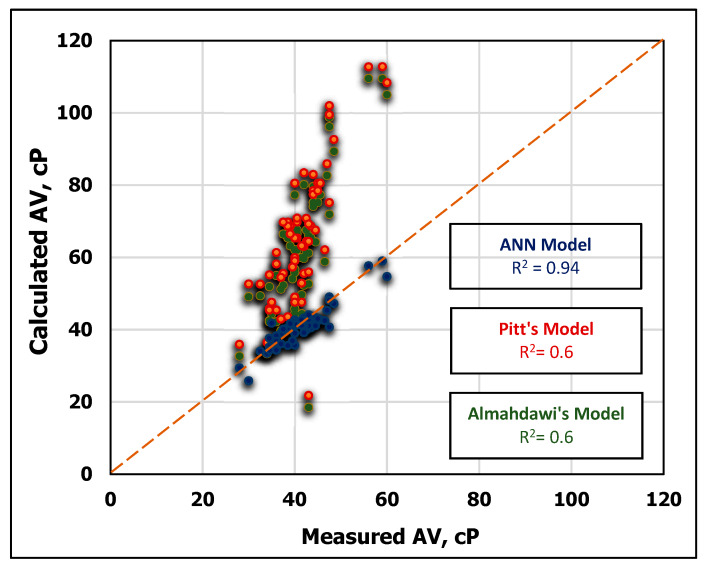
Comparison of predicted AV using Pitt’s [54], Almahdawi’s et al. [55] and the developed ANN models.

**Figure 12:**
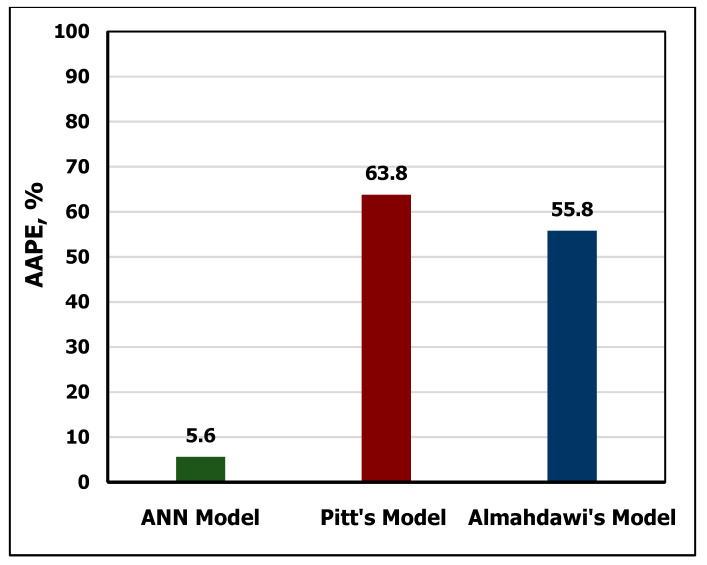
Comparison between the developed (AV) ANN model and the previous approaches.

The developed AV correlation was validated by comparing its results with the previously published approaches. To verify the developed model, the testing data of MD and FV were utilized to estimate AV using the two previous models in Equations (13) and (14) and the newly developed ANN-AV model. The obtained results showed that the ANN model outperformed with R^2^ of 0.94 compared to R^2^ of 0.63 for both Equation (13) and Equation (14), respectively as shown in Figure 11 and Figure 12. The superiority of the developed ANN model over the other models can be also indicated in Figure 12, which shows that the error of the developed ANN model was only 3.4% AAPE compared with 63.8% AAPE for Equation (13) and 55.8% for Equation (14). 

## 4. Conclusions

Actual field measurements (200 data points) were used to build new ANN models to predict the rheological parameters of high-bentonite mud (HBM) while drilling. The following conclusions can be drawn based on the findings of this study:

1. The new ANN models can predict the rheological parameters (PV, Y_P_, and AV) for HBM while drilling based on MD and FV with high accuracy (R-value was greater than 0.90 and AAPE was less than 6%).
2. The optimized models were developed using a network of a single hidden layer with 20 neurons processed by Levenberg-Marquardt algorithm. The optimum training rate was 0.12 for developing the ANN models. Tan-sigmoidal was used as a transfer function to get the best results with the linear function as an activation function for the output layer.
3. The developed ANN-based empirical equations provide a practical way to estimate the rheological parameters of HBM directly without requiring any special programs or compilers.
4. The developed ANN-based model for the apparent viscosity outperformed the previously published correlations.

## Figures and Tables

**Table 1:** HBM additives.

Additive	Range/Unit	Uses
Bentonite	40–50 lb/bbl	Viscosifier Fluid loss control
Enviro-thin	1–3 lb/bbl	Dispersant Shear strength reduce/deflocculant
Caustic Soda (NaOH)	0.2–0.5 lb/bbl	pH adjustment
Starch	1–3 lb/bbl	Fluid loss control
Bactcide	0.01–0.03 gal/bbl	Antibacterial/Biocide

**Table 2:** HBM formulation prepared in the laboratory for the experimental investigation.

Item	Quantity	Unit
Water	340	g
Bentonite	40	g
Caustic Soda	0.5	g
Dispersant	3	g
Starch	3	g

**Table 3:** Statistical analysis of the obtained data for HBM.

Statistical Quantity	MD	FV	PV	Y_p_	AV
Mean	67.6	85.4	25.2	30.3	40.3
Median	68.0	82.0	25.0	30.0	40.0
Mode	68.0	85.0	25.0	26.0	40.0
Range	9.0	105.0	45.0	26.0	56.0
Minimum	64.0	45.0	11.0	20.0	23.0
Maximum	73.0	150.0	56.0	46.0	79.0
Standard Deviation	1.4	19.6	8.0	5.3	9.5
Kurtosis	6.2	1.1	1.7	0.8	2.0
Skewness	1.5	1.0	0.9	0.7	0.9

**Table 4:** Sample of the filtered HBM field data.

No.	MD (lb/ft^3^)	FV (s)	PV (cP)	Y_p_ (lb/100 ft^2^)	AV (cP)
1	68	60	11	28	25
2	64	135	13	20	23
3	69	80	12	27	25.5
4	73	70	15	30	30
5	67	75	16	22	27
6	68	59	11	27	24.5
7	69	85	40	25	52.5
8	66	150	45	40	65
9	68	110	31	31	46.5
10	67	90	31	36	49
11	68.5	76	29	28	43
12	67	105	29	36	47
13	73	108	40	28	54
14	67	92	40	36	58
15	68.5	75	32	26	45

**Table 5:** Reference rheological properties of field high-bentonite mud formulation.

Property	Range	Unit	Remarks
Mud weight	67	lb/ft^3^	Mud balance measurement
Marsh Funnel Viscosity	75–120	s	Qualitative measurement
pH	9.5–10.5		Alkalinity measurement
Initial Gel Strength	15–27	lb/100 ft^2^	10-senocd rheometer measurement
10-min Gel Strength	25–75	lb/100 ft^2^	10-min rheometer measurement
30-min Gel Strength	26–80	lb/100 ft^2^	30-min rheometer measurement
Yield Point	25–50	lb/100 ft^2^	Rheometer measurement
Plastic Viscosity	10–60	cP	Rheometer measurement
Filtrate Volume	8–10	cm^3^/30 min	Filter Press measurement

## Nomenclature

HBM	High-bentonite mud
MD	Mud density
FV	Marsh funnel viscosity
Yp	yield point
PV	Plastic viscosity
AV	Apparent viscosity
ECD	Equivalent circulating density
AAPE	Average absolute percentage error
R	Correlation coefficient
tansig	Hyperbolic tangent sigmoid transfer function
logsig	Log-sigmoid transfer function
elliotsig	Elliot symmetric sigmoid transfer function
radbas	Radial basis transfer function
purelin	Linear transfer function
Tribas	Triangular basis transfer function
hardlim	Hard-limit transfer function
satlin	Saturating linear transfer function
trainlm	Levenberg-Marquardt backpropagation
trainrp	Resilient backpropagation
trainbr	Bayesian regularization
trainc	Cyclical order incremental update
traingda	Gradient descent with adaptive learning rule backpropagation
traingd	Gradient descent backpropagation

## Appendix A

The inputs should be normalized between [−1 and 1] to calculate the values MD_n_ and FV_n_, then these values can be used within the developed set of equations to estimate the normalized values of Y_p_, PV, and AV using Equations (7), (9), and (11), respectively. The normalization can be done using the two-point slope form as shown in the following equation:

(A1) $$\frac{Y-Y_{\min}}{Y_{\max}-Y_{\min}}=\frac{X-X_{\min}}{X_{\max}-X_{\min}}$$

where Y is the normalized value of the input parameter, Y_min_ = −1, Y_max_ = 1, X is the input parameter (MD or FV), X_min_ is the minimum value of the input parameter, and X_max_ is the maximum value of the input parameter.

(A2) $$Y=2\times \left(\frac{X-X_{\min}}{X_{\max}-X_{\min}}\right)-1$$

## Appendix B

**Table A1:** Extracted constants for estimating the normalized $Y_{p_{n}}$ in Equation (7).

i	$W_{1_{i,j}}$		$W_{2_{i}}$	$b_{1,i}$	$b_{2}$
	j = 1	j = 2			
1	−6.438	−1.722	−0.976	5.256	−0.646
2	−3.871	−5.183	−1.309	5.189	
3	1.196	8.918	1.450	−4.763	
4	5.324	3.405	−0.843	−5.262	
5	−4.281	−3.710	0.938	5.971	
6	−0.898	8.725	−1.511	−4.598	
7	−6.191	0.259	0.783	3.726	
8	−6.592	−0.724	0.333	0.379	
9	7.256	1.198	0.466	−1.105	
10	5.796	1.522	0.252	3.285	
11	5.534	−1.909	−0.561	3.017	
12	−5.377	−10.789	−0.131	−4.180	
13	−0.876	−10.431	−0.192	0.002	
14	5.633	−2.006	−1.256	3.742	
15	5.424	−3.472	0.839	3.876	
16	5.851	−7.147	0.596	3.011	
17	−4.425	2.739	−0.628	−5.963	
18	−9.930	−3.096	0.126	−1.936	
19	−5.837	12.148	0.667	−5.094	
20	−9.246	1.508	−0.581	−5.767	

**Table A2:** Constants for estimating the normalized PV_n_ in Equation (9).

i	$W_{1_{i,j}}$		$W_{2_{i}}$	$b_{1,i}$	$b_{2}$
	j = 1	j = 2			
1	3.457	4.020	0.190	−7.611	0.811
2	−5.002	3.575	1.172	6.281	
3	−5.545	−3.368	−0.390	5.267	
4	−6.538	−1.564	−0.718	2.211	
5	5.248	−2.050	−0.798	−4.631	
6	−2.253	3.617	−0.827	3.458	
7	−3.930	6.908	0.134	2.085	
8	−9.102	5.892	0.675	3.095	
9	−1.800	−7.060	0.258	−2.057	
10	−1.567	4.346	0.584	−3.198	
11	10.680	−7.060	−0.188	1.384	
12	2.985	−0.144	0.230	−0.355	
13	−3.206	1.819	−0.608	0.834	
14	−1.983	−0.408	0.274	−0.381	
15	−5.065	1.269	−0.124	−2.345	
16	−0.031	−9.201	0.150	−4.596	
17	−4.567	−5.151	−0.424	−2.586	
18	6.310	0.927	−1.990	6.763	
19	2.492	5.563	0.158	6.596	
20	−6.164	0.435	−1.146	−5.166	

**Table A3:** Constants for estimating the normalized ${\mathrm{AV}}_{n}$ in Equation (11).

i	$W_{1_{i,j}}$		$W_{2_{i}}$	$b_{1,i}$	$b_{2}$
	j = 1	j = 2			
1	7.678	−2.892	0.384	−4.868	−0.365
2	3.632	−5.384	−1.561	−6.839	
3	4.647	4.557	0.499	−2.401	
4	−5.234	−2.113	−0.325	5.115	
5	6.199	0.612	0.510	−4.399	
6	−6.369	3.674	1.956	2.124	
7	−2.347	5.874	−0.911	−2.496	
8	0.733	−6.371	1.034	−5.309	
9	−8.594	3.012	−1.277	1.524	
10	1.460	−3.995	−1.316	1.886	
11	7.201	−9.123	−0.219	−0.057	
12	7.542	6.624	−0.229	−1.789	
13	−1.736	−7.167	0.574	−1.700	
14	−2.995	−0.354	0.103	−10.009	
15	5.760	3.549	0.693	2.579	
16	−8.152	0.364	0.029	−2.345	
17	−2.961	5.941	0.593	1.817	
18	0.420	7.370	−0.733	3.554	
19	−8.938	−2.516	−0.356	−6.634	
20	−4.404	−1.935	−0.346	−9.442	
